# Supplementary material for: Performances of Limited Area Models for the WORKLIMATE Heat–Health Warning System to Protect Worker’s Health and Productivity in Italy
Source: Int J Environ Res Public Health. 2021 Sep 21;18(18):9940. doi: 10.3390/ijerph18189940 (PMC8466979; doi:10.3390/ijerph18189940)
Supplement: Supplementary file 1 [file ijerph-18-09940-s001.zip › ijerph-1346203-supplementary.pdf]

Supplemetary material

Table S1. Day 3-WBGT-shade average categorical skill scores for the 12-18 time slot for each geographical macro-areas. In the "northern inland plain areas", Bolzano values were not included in the average.

	A			B			C		
Model	BOL	MOL_E	MOL_G	BOL	MOL_E	MOL_G	BOL	MOL_E	MOL_G
Data	1902	1116	594	1895	1111	590	1887	1190	723
HR	80.1	75.2	73.5	78.8	77.4	76.1	73.4	77.0	77.1
CSI	75.0	71.4	68.0	74.9	75.4	72.0	68.0	74.7	72.9
POD1	78.1	75.2	80.9	81.7	78.0	81.9	78.1	78.2	81.1
POD2	87.5	92.9	89.6	75.2	86.8	85.7	63.4	86.1	88.0
POD3									
POD1x	95.4	96.4	96.5	94.9	96.9	95.0	88.2	95.3	94.1
POD2x	87.8	93.5	89.9	75.3	87.4	86.1	63.5	86.2	88.1
NA	10.5	12.8	9.8	13.8	17.5	15.8	24.0	26.0	22.5
FA	22.6	43.0	46.9	19.6	46.7	45.9	14.3	46.2	44.1
NA*	2.5	1.9	1.8	2.3	1.4	2.6	5.4	2.4	3.1
FA*	5.9	9.7	15.0	3.7	6.2	10.6	2.9	6.5	9.8
RLO 1	52.9	53.9	52.9	46.2	49.9	53.8	48.0	51.9	54.7
RLO 2	20.8	23.3	14.9	34.2	35.5	21.1	32.3	33.0	20.8
RLO 3	0.0	0.0	0.0	0.4	0.4	0.2	0.3	0.3	0.1
RLP 1	49.6	51.5	59.1	50.3	50.0	58.0	53.1	51.6	56.7
RLP 2	27.4	33.3	21.9	31.8	40.2	25.0	25.0	37.5	25.6
RLP 3	0.1	0.2	0.1	0.0	0.4	0.2	0.0	0.0	0.0

Model: BOL, BOLAM initialized on the GFS; MOL-E, MOLOCH initialized on the ECMWF; MOL-G, MOLOCH initialized on the ECMWF; Data, sample size; HR, hit Rate (%); CSI, critical success index (%); POD1, probability of risk level 1 detection (%); POD2, probability of risk level class 2 detection (%); POD3, probability of risk level 3 detection (%); POD1x, probability of risk level 1 or higher class detection (%); POD2x, probability of risk level 2 or higher class detection (%); NA, lack alarm (%); FA, false alarm (%); NA*, normalized lack alarm (%); NA*, normalized false alarm (%); RLO1, risk level 1 observed (%); RLO2, risk level 2 observed (%); RLO3, risk level 3 observed (%); RLP1, risk level1 predicted (%); RLP2, risk level2 predicted (%); RLP3, risk level 3 predicted (%); empty cell, it was not possible to calculate the indicator due to the lack of data observed or predicted by the model for at least one location.

Table S2. Day 3-WBGT-sun average categorical skill scores for the 12-18 time slot for each geographical macro-areas. In the "northern inland plain areas" (A), Bolzano values were not included in the average.

	A			B			C		
Model	BOL	MOL_E	MOL_G	BOL	MOL_E	MOL_G	BOL	MOL_E	MOL_G
Data	1884	1105	592	1892	1109	589	1886	1105	593
HR	76.7	72.9	72.2	79.9	78.9	76.7	71.5	75.8	75.5
CSI	72.5	69.9	68.4	77.6	78.2	75.3	68.2	74.9	74.1
POD1	69.9	70.2	74.2	74.7	75.5	80.4	71.7	76.4	80.7
POD2	86.5	86.6	87.8	88.1	91.4	90.0	76.3	86.2	85.4
POD3	53.9	60.3	44.0	17.7	34.1	34.0	9.0	40.0	52.0
POD1x	93.4	94.9	95.2	94.5	96.8	96.8	85.5	93.9	93.7
POD2x	90.0	93.6	92.7	88.7	93.6	92.2	77.0	88.7	87.6
NA	13.7	17.0	17.9	15.5	29.8	23.5	28.6	37.9	35.0
FA	32.9	56.0	59.4	26.8	66.8	63.5	21.0	61.4	60.6
NA*	2.4	1.6	1.8	1.7	0.9	1.4	4.5	1.6	2.5
FA*	6.3	8.7	11.8	3.8	5.2	8.7	3.0	4.8	7.6
RLO 1	40.9	43.8	49.9	35.4	40.0	50.8	35.1	40.2	49.7
RLO 2	36.2	36.4	25.4	49.1	49.7	33.7	47.5	48.0	34.8
RLO 3	0.6	0.9	0.6	1.7	2.1	1.2	4.3	3.9	2.6
RLP 1	37.7	41.2	49.9	35.9	38.5	52.4	39.5	40.7	52.0
RLP 2	42.1	43.4	34.1	52.0	56.0	39.7	45.3	52.7	39.1
RLP 3	1.7	3.5	2.0	0.4	1.5	1.0	0.5	1.9	1.3

Model: BOL, BOLAM initialized on the GFS; MOL-E, MOLOCH initialized on the ECMWF; MOL-G, MOLOCH initialized on the ECMWF; Data, sample size; HR, hit Rate (%); CSI, critical success index (%); POD1, probability of risk level 1 detection (%); POD2, probability of risk level class 2 detection (%); POD3, probability of risk level 3 detection (%); POD1x, probability of risk level 1 or higher class detection (%); POD2x, probability of risk level 2 or higher class detection (%); NA, lack alarm (%); FA, false alarm (%); NA*, normalized lack alarm (%); NA*, normalized false alarm (%); RLO1, risk level 1 observed (%); RLO2, risk level 2 observed (%); RLO3, risk level 3 observed (%); RLP1, risk level1 predicted (%); RLP2, risk level2 predicted (%); RLP3, risk level 3 predicted (%); empty cell, it was not possible to calculate the indicator due to the lack of data observed or predicted by the model for at least one location.

Table S3. Average values of Mean error, mean absolute error and root mean square error of the Day 3-WBGT-sun predicted for the 12-18 time slot for the three geographical macro-areas. The scores were calculated both considering all its hourly data and the its maximum value. In the "northern inland plain areas" (A), Bolzano values were not included in the average.

	A			B			C		
Model	BOL	MOL_E	MOL_G	BOL	MOL_E	MOL_G	BOL	MOL_E	MOL_G
MAE	1.5	1.6	1.8	1.2	1.3	1.3	1.5	1.3	1.3
RMSE	2.0	2.2	2.3	1.6	1.6	1.7	1.9	1.7	1.7
ME	0.8	1.2	1.4	0.1	0.6	0.7	-0.4	0.5	0.7
Data	1896	1113	592	1892	1109	589	1887	1104	593
MAEmax	1.2	1.4	1.6	1.2	1.2	1.3	1.5	1.3	1.2
RMSEmax	1.7	1.9	2.0	1.5	1.5	1.6	1.8	1.6	1.6
MEmax	0.6	1.1	1.4	-0.1	0.5	0.7	-0.6	0.6	0.8
Datamax	317	186	99	317	186	99	315	184	99

Model: BOL, BOLAM initialized on the GFS; MOL-E, MOLOCH initialized on the ECMWF; MOL-G, MOLOCH initialized on the ECMWF; MAE, mean absolute error; RMSE, root mean square error; ME, mean error; Data, sample size; MAEmax, mean absolute error of the maximum time slot value; RMSEmax, root mean square error of the maximum time slot value; MEmax, mean error of the maximum time slot value; Datamax, maximum value sample size.

Table S4. Average values of Mean error, mean absolute error and root mean square error of the Day 3-WBGT-shade predicted for the12-18 time slot for the three geographical macro-areas. The scores were calculated both considering all its hourly data and the its maximum value. In the "northern inland plain areas" (A), Bolzano values were not included in the average.

	A			B			C		
Model	BOL	MOL_E	MOL_G	BOL_G	MOL_E	MOL_G	BOL_G	MOL_E	MOL_G
MAE	1.2	1.3	1.4	1.1	1.1	1.1	1.4	1.1	1.1
RMSE	1.6	1.7	1.8	1.4	1.4	1.4	1.8	1.5	1.4
ME	0.5	1.0	1.1	-0.1	0.3	0.5	-0.6	0.3	0.5
Data	1902	1116	594	1892	1107	587	1891	1106	594
MAEmax	1.1	1.3	1.4	1.1	1.1	1.1	1.4	1.1	1.1
RMSEmax	1.4	1.7	1.7	1.4	1.3	1.4	1.7	1.4	1.4
MEmax	0.4	1.1	1.3	-0.3	0.3	0.4	-0.8	0.4	0.6
Datamax	317.0	186.0	99.0	316.2	185.2	98.2	315.3	184.4	99.0

Model: BOL, BOLAM initialized on the GFS; MOL-E, MOLOCH initialized on the ECMWF; MOL-G, MOLOCH initialized on the ECMWF; MAE, mean absolute error; RMSE, root mean square error; ME, mean error; Data, sample size; MAEmax, mean absolute error of the maximum time slot value; RMSEmax, root mean square error of the maximum time slot value; MEmax, mean error of the maximum time slot value; Datamax, maximum value sample size.
